# Health and health-related behaviours in refugees and migrants who self-identify as sexual or gender minority – A National population-based study in Sweden

**DOI:** 10.1016/j.eclinm.2022.101641

**Published:** 2022-09-01

**Authors:** Erica Mattelin, Frida Fröberg, Laura Korhonen, Amal R. Khanolkar

**Affiliations:** a Barnafrid, Swedish National Center on Violence Against Children, Department of Biomedical and Clinical Sciences, Linköping University, Linköping, Sweden; b Center for Social and Affective Neuroscience, Department of Biomedical and Clinical Sciences, Linköping University, Linköping, Sweden; c Department of Child and Adolescent Psychiatry and Department of Biomedical and Clinical Sciences, Linköping University, Linköping, Sweden; d Department of Global Public Health, Karolinska Institutet, Stockholm, Sweden; e Department of Population Health Sciences, School of Life Course & Population Sciences, King's College London, London, United Kingdom

**Keywords:** Ethnicity, Migrant, Refugee, Sexual minority, Gender minority, LGBTQ+, Transgender health, Mental health, Inequalities, Sweden

## Abstract

**Background:**

To examine health and health-related behaviors in migrant and refugee individuals who identify as sexual or gender minority, and in comparison to their heterosexual peers.

**Methods:**

The study included 168,952 individuals (aged 16–84 years, males: 45·9%, sexual or gender minorities: 3·1%) who answered the Swedish National Public Health Survey in 2018 and 2020. Participants were grouped into Swedish-and Western-born (White) heterosexual, White sexual- or gender minority, migrant heterosexual, migrant sexual- or gender minority, refugee heterosexual, and refugee sexual- or gender minority. Outcomes included mental health (for example suicidal ideation, wellbeing), general health, risky behaviors (risk alcohol use, risk gambling, and substance use), and experiences of violence. Associations between 1) sexual- or gender -ethnic identities and 2) gender-ethnic identities and all outcomes were analyzed using logistic and linear regression adjusting for sex, age, and educational level.

**Findings:**

Being a sexual- or gender minority, regardless of ethnic minority status, was associated with worse general health and mental ill-health compared to heterosexual peers including suicidal ideation in refugee sexual- or gender minority individuals (OR 2·42, 95 % CI 1·44–4·08). Ethnic minorities (heterosexual and sexual- or gender minority migrants and refugees) had lower odds of drug and risk alcohol use compared to White heterosexual peers but higher odds of risk gambling (1·88, 1·49–2·37 for refugee heterosexuals**)**. Transgender refugees had high odds for risk gambling (8·62, 1·94–38·40) and exposure to physical violence (7·46, 2·97–18·70).

**Interpretation:**

In this national population-based study, sexual and gender minority individuals have worse mental and general health regardless of ethnic minority status. We did not find evidence for worse health in sexual- or gender minority refugees in comparison to migrant, and White sexual- or gender minorities and their heterosexual peers. Transgender individuals (White and ethnic minority) experienced significantly higher levels of physical violence. Public health policy should emphasize preventive measures to reduce exposure to violence and discrimination in sexual- and gender minority individuals, increase access and use of mental healthcare services and sensitise healthcare professionals about higher rates of health and related issues faced by sexual- and gender minority individuals including those with multiple minority identities.

**Funding:**

We received no external funding for this study and hence the funder had no role in the study design, data collection, data analysis, data interpretation, writing of the manuscript and the decision to submit.

**Research in context**

**Evidence before this study**

We searched PubMed in June 2021 for search terms related to ‘sexual and gender minorities’ AND ‘refugees or migrants’, AND ‘mental health, health, and other health-related aspects’. Retrieved studies showed limited evidence for poorer mental health in refugee, and transgender sexual minorities, and these studies were small in size. We found no studies using a nationally representative sample combining refugees and migrant sexual- and gender minorities, including heterosexual comparator groups. We also went through recent reports and updates on the field. We finally conducted a back- and forward search from key articles.

**Added value of this study**

To our knowledge, this is the first nationally representative study (*N*=164,118) to separately examine health in both refugee and migrant sexual- and gender minority individuals, and in comparison, to their heterosexual and cisgender peers. It is also the first national study of its kind to examine health in transgender refugee and migrant individuals. Our study provides substantial evidence for poorer health and exposure to violence in diverse sexual- and gender minority groups. While more research is needed, our study shows the need for public health personnel to be aware of potentially worse health and adverse experiences in sexual and gender minority individuals, including refugees.

**Implications of all the available evidence**

Our findings emphasize that current policies tackling health in refugees and sexual- and gender minorities still are not enough. This knowledge is vital for countries like Sweden that have received substantial refugees during the past decade. There is also a need for more research on the mechanisms that lead to poorer health outcomes in these individuals, and comprehensive longitudinal data that is currently lacking.

Alt-text: Unlabelled box

## Introduction

In 2019, 272 million, or 3·5% of the world's population crossed international borders.^1^ At the same time, 80 million individuals were displaced. Of these, 20·7% were refugees and 4·1% were asylum-seekers. The remaining 75% includes individuals who are categorized as internally displaced.^2^ The reasons for migrating from one's home country are diverse. In the most recent decade, the world has witnessed a substantial increase in asylum seekers due to political conflicts, widespread violence, economic crisis, and climate change reflecting an increasingly complex world.^1^ Sweden received 780,215 asylum claims between 2000-2022^3^ and was for a period, a country with one of the highest numbers of asylum-claims per capita in the European Union (15,931/1,000,000 in 2015).^4^ Most asylum-seekers (45%) migrated from Syria, Afghanistan, Iraq and Somalia.^3^ During the same period (2000-2021), 1,629,895 resident permits were granted to migrant individuals for reasons other than seeking asylum.^5^ In 2018, the majority (52%) of a total 41,050 work permits allocated to non-EU countries were given to individuals who originated from India, Thailand, Ukraine, Iraq and China.

Substantial research shows that migrants (i.e., individuals who migrate voluntarily for work, education, and family reunification)^6^ in high-income countries have better health compared to their host population.^7^ However, the reverse is observed in refugees. Being a refugee is associated with increased short- and long-term risk of poorer mental and physical health, even several years after settlement in the host country.^8^^,^^9^

Despite considerable improvement in sexual and gender minority (SGM) rights and greater societal acceptance, substantial evidence shows that SGM individuals have increased risk for mental ill-health and poorer physical health,^10^^,^^11^ are more likely to engage in adverse health-related behaviors (for example, substance use and risky sex),^12^ and experience the co-occurrence of both.^13^ They are also at increased risk of exposure to violence.^14^ However, this evidence is largely based on studies including White individuals and mostly originate from the USA. Health in individuals who identify as being both ethnic minority and sexual/gender minority (EM-SGM) i.e., dual minority identities are limited. These studies on health in EM-SGM individuals are often smaller in size, focusing on select ethnic minorities (EM) and rarely use national population-based data, especially in the European context.^15^^,^^16^ These studies also mostly originate from the USA, with a different healthcare system and distribution of ethnic minority groups making it incomparable to the European context.^17^, ^18^, ^19^ Further, these studies show contradictory results. While most show evidence for worse mental health in EM-SGM individuals compared to their White-SM peers, others have found the opposite.^18^^,^^20^ Additionally, many of the studies have focused on younger EM-SGM individuals.^21^ Refugees who identify as SGM could be at an even higher risk for adverse health because of past experiences of violence and mental ill-health associated with migration combined with racism and stigma in their home and host countries. There is ample evidence for discrimination including racism towards refugee and migrant individuals, including those of Middle-Eastern background in Sweden (for example, in accessing the labour market and housing).^22^^,^^23^

The few studies that have investigated health in the SGM refugee individuals have been limited to small-sample quantitative studies (<500 participants) and qualitative studies.^24^^,^^25^ To our knowledge, no study has examined health in SGM refugees in a nationally representative sample including comparisons with both heterosexual and SGM White and migrant populations. Including these different groups enables comparing risks between these groups of migrants who have migrated for potentially different reasons. Limited research shows that transgender individuals have increased risks for discrimination and mental ill-health compared to cisgender peers. However, the evidence on health in EM and refugee transgender individuals is limited due to very small numbers and lack of data.^24^^,^^26^

The intersectional framework theory is often put forth to explain adverse health in those individuals with ≥2 minority identities. It hypothesizes that the ‘intersection’ between multiple subordinated minority identities is associated with different forms of discrimination within existing social hierarchies further perpetuating inequalities.^27^ These multiple minority identities need to be investigated jointly to fully understand their impact on health inequalities, and it is crucial to investigate them including all identity variables in the same model and testing for interactions between them and the outcomes of interest.^28^ Identifying and examining health in marginalized groups is essential as it can aid in designing interventions that can be implemented earlier when refugees and migrants arrive in a host country. Mental health can also affect integration^29^ resulting in long-term consequences (e.g., future financial situation and educational attainment).^30^

The main aim of this study was to use an intersectional framework to examine whether individuals with dual ethnic (refugee *or* migrant) and sexual minority identities were more likely to have poorer mental and general health, worse health-related behaviours and exposure to violence in comparison, to their heterosexual Swedish- and western-born cisgender peers, in a national population-based sample. Additionally, we also examined whether these associations differed between transgender and cisgender individuals with refugee or migrant background compared to Swedish- and western-born peers.

## Methods

### Study population and design

The Swedish National Public Health survey “Health on Equal Terms” is conducted by The Public Health Agency of Sweden to examine change in health over time. The survey collects information on sociodemographic data, mental and physical health, health-related behaviors, and living conditions. It was initiated in 2004 and is conducted every two years (online since 2007) with different samples each year. It includes a nationally representative sample of individuals aged 16-84 years (the selected frame) drawn from the National Population Register. The survey is available in both Swedish and English and can be answered using either the online or paper questionnaires. For this study, we retrospectively merged the 2018 and 2020 survey responses. Our sample consisted of 168,952 individuals with a participation rate of 42·1% in 2018 and 42·3% in 2020.^31^^,^^32^ While 157,414 individuals had data on ethnicity, sexuality and all covariates, 164,118 individuals had data on ethnicity, gender identity and all covariates and were included in the analyses. Missing data was <5% for all variables. While age and sex at birth was available for all participants, proportions of missing data for all other variables ranged from 0.6 to 1.8% (Table 1). We chose the 2018 and 2020 surveys as they provide information on the country of origin, making it possible to create our exposure variable of interest.

**Table 1:** Descriptive characteristics by ethnic and sexual/gender identities of 168,952 individuals who took part in the Swedish national public health survey in 2018 and 2020.

Covariates and outcomes	Dual sexual/gender and ethnic identity indicator														
	White heterosexual (*N =* 143 694)		White Sexual/gender minority (*N =* 4688)		Migrant heterosexual (*N =* 4300)		Migrant Sexual/gender minority (*N =* 285)		Refugee Heterosexual (*N =* 4194)		Refugee sexual/gender Minority (*N =* 253)		Test for Difference^a^	Missing	
	*N*	%	*N*	%	*N*	%	*N*	%	*N*	%	*N*	%		*N*	%
**Sex at birth** Male	66440	46·2	1786	38·1	2307	53·7	174	61·1	1710	40·8	114	45·1	<0·001	0	0
Female	77254	53·8	2902	61·9	1993	46·3	111	38·9	2484	59·2	139	54·9			
**Age (years)** 16-17	2018	1·4	171	3·6	109	2·5	5	1·8	63	1·5	4	1·6	<0·001	0	0
18-29	15377	10·7	1603	34·2	862	20·0	54	18·9	472	11·3	67	26·5			
30-45	26262	18·3	1306	27·9	1407	32·7	107	37·5	1561	37·2	121	47·8			
46-64	47069	32·8	977	20·8	1499	34·9	98	34·4	1488	35·5	50	19·8			
65+	52968	36·9	631	13·5	423	9·8	21	7·4	610	14·5	11	4·3			
**Foreign background** Born outside Sweden	8798	6·1	338	7·2	4238	100	282	100	4095	100	239	100	<0·001	1060	0·6
Born in Sweden with two parents born abroad	3232	2·2	168	3·6	12	0	0	0	0	0	0	0			
Born in Sweden with one parent born abroad	9240	6·4	455	9·7	5	0	0	0	0	0	0	0			
Born in Sweden with two Swedish parents	122236	85·2	3710	79·4	0	0	0	0	0	0	0	0			
**Educational level**^a^ High	49533	34·7	1634	35·2	1277	31·5	76	28·3	1659	40·8	93	39·2	<0·001	1951	1·2
Medium	57814	40·5	1303	28·1	1270	31·4	81	30·1	1239	30·5	65	27·4			
Low	24164	16·9	458	9·9	969	23·9	76	28·3	860	21·2	39	16·5			
Too young to estimate	11272	7·9	1241	26·8	533	13·2	36	13·4	305	7·5	40	16·9			
**General health** Good or very good	101350	71·2	3017	64·7	3021	70·8	196	69·3	2989	71·8	191	76·1	<0·001	1720	1·0
Moderate	33720	23·4	1228	26·4	875	20·5	67	23·7	897	21·5	44	17·5			
Bad or very bad	7722	5·4	415	8·9	370	8·7	20	7·1	278	6·7	16	6·4			
**Mental well-being** Median (25^th^/75^th^ percentile))	28	27/31	27	24/30	28	25/31	28	24/32	28	25/31	28	25/31		3027	1·8
**Mental health** Mental ill-health^b^	15118	10·6	1162	24·8	789	18·5	66	23·3	570	13·7	53	21·0	<0·001	1229	0·7
**No**	127774	89.4	3818	75.2	3483	81.5	217	76.7	3592	863	199	790			
**Suicidal ideation** Yes, ever	14185	9·9	1668	35·8	461	10·8	47	16·7	475	11·4	62	24·7	<0·001	1415	0·8
No	128539	90·1	2995	64·2	3795	89·2	234	83·3	3687	88·6	189	75·3			
**Suicide attempts** Yes, ever	3803	2·7	654	14·0	187	4·4	26	9·3	178	4·3	30	11·9	<0·001	1398	0·8
No	138935	97·3	4007	86·0	4071	95·6	255	90·7	3980	95·7	222	88·1			
**Health-related behaviours in the previous 12 months**															
**Risk gambling**^c^													<0·001	1998	1·2
Yes	4254	3·0	171	3·7	283	6·7	27	9·6	246	5·9	13	5·2			
No	138059	97·0	4485	96·3	3969	93·3	255	90·4	3909	94·1	237	94·8			
**Substance use**^d^													<0·001	775	0·5
Yes	2487	1·7	373	8·0	71	1·7	15	5·3	71	1·7	12	4·8			
No	140693	98·3	4298	92·0	4208	98·3	269	94·7	4112	98·3	240	95·2			
**Risk alcohol use**															
Yes	22347	15·6	1037	22·2	184	4·3	17	6·0	315	7·5	31	12·3	<0·001	526	0·3
No	121078	84·4	3644	77·8	4101	95·7	267	94·0	3874	92·5	1238	87·7			
**Exposure to any kind of violence or discrimination**															
Yes	29076	20·3	1998	42·7	1171	27·3	85	29·6	1178	28·2	89	35·3	<0·001	934	0·6
No	114079	79·7	2676	57·3	3116	72·7	199	70·1	3001	71·8	163	64·7			
**Exposure to threats**															
Yes	5306	3·7	447	9·6	272	6·4	30	10·8	220	5·3	19	7·6	<0·001	2027	1·2
No	137004	96·3	4195	90·4	3977	93·6	248	89·2	3938	94·7	231	92·4			
**Exposure to discrimination**															
Yes	25518	17·9	1820	39·0	983	23·0	52	18·4	1008	24·2	80	31·7	<0·001	1600	0·9
No	117272	82·1	2846	61·0	3283	77·0	230	81·6	3154	75·8	172	68·3			
**Exposure to physical violence**															
Yes	3391	2·4	274	5·1	164	3·8	26	9·2	174	4·2	15	6·0	<0·001	1411	0·8
No	139509	97·6	4391	94·9	4105	96·2	257	90·8	3997	95·8	236	94·0			

a p values are for a test of means or equal proportions.

b Mental health based on the General Health questionnaire (GHQ) used in 2018 and the Kessler-6 used in 2020. 3 Low (No education or Elementary school, primary school, or similar) Medium (Two years of upper secondary school or high school or 3–4 years of upper secondary school or high school) High (Some higher education/ University or college, less than 3 years/ University or college, 3 years or more).

c Score of more than one on PSGI.

d Any use of drugs during the last year.

### Variables of interest

#### Sexual identity and ethnicity

In the survey, participants were asked the following questions on sexual and gender identities: “How would you define your sexual identity?” and “Are you or have you been a transgender person?” (Supplemental Table 1). Based on responses, we categorized participants into 1. Heterosexual, and 2. Sexual and/or gender minority (homosexual, bisexual, and transgender). Participants who answered ‘don't know’ or ‘else’ to both questions were excluded. In consultation with the Swedish Migration Board, ethnicity was based on year of birth, year of immigration, and country of birth obtained from the Total Population register, and refugee status from the register STATIV, a database for integration studies (Supplemental Table 2). This was necessary as Sweden does not collect self-identified ethnicity. Individuals were categorized into: 1. Swedish- and Western-born, hereafter referred to as White, 2. migrant, and 3. refugee/asylum seeker groups. Migrant and refugee individuals constitute ethnic minorities.The category ‘White’ included individuals born in Sweden, the Nordic countries, the EU, Oceania, the USA after 1945. The category refugees includes both former refugees settled in Sweden and current refugees. However, the vast majority of individuals in this category are former refugees.

#### Sexuality & migration indicator (study exposure)

Our exposure of interest was created by combining the sexual identity and ethnicity variables resulting in one variable with the following six categories. 1. White heterosexual, 2. White sexual and/or gender minority (White-SGM) 3. Migrant heterosexual 4. Migrant sexual and/or gender minority (migrant-SGM) 5. Refugee heterosexual 6. Refugee sexual and/or gender minority (refugee-SGM) [Supplemental Table 1]. This approach allows for estimating risk in all three categories of sexual/gender- and ethnic-identities compared to the reference White heterosexual group and incorporates testing for interaction effects between the different categories (and consequently an ‘intercategorical’ approach in examining intersectionality between ethnic and sexual identities and their impact on health).^28^

Further, we created two other exposure variables used in sensitivity analysis: A. To examine differences between transgender and cisgender individuals; 1. White cisgender 2. White transgender 3. Migrant cisgender 4. Migrant transgender 5. Refugee cisgender 6. Refugee transgender (Supplemental Table 1). B. To examine differences between SM identities; 1. White heterosexual 2. White bisexual 3. White homosexual 4. Migrant heterosexual 5. Migrant bisexual 6. Migrant homosexual 7. Refugee heterosexual 8. Refugee bisexual 9. Refugee homosexual.

### Outcomes

All outcomes in this study were answered by participants who took part in the survey. Detailed information on the original questions and how these were used in the analysis are listed in Supplemental Table 1.

### General health

Information about general health was assessed by the question “How would you rate your general health?”. Responses were dichotomized into good vs. poor.

### Mental ill-health

Mental ill-health (symptoms of anxiety and depression) was assessed by the General Health Questionnaire (GHQ-5) in 2018 and the Kessler-6 scale in 2020, due to a change in mental-health assessment between the two surveys. To obtain a measure for psychological distress for GHQ-5, a sum index is calculated based on the first five questions.^31^^,^^33^ Participants with a value of 1 are defined as having reduced mental wellbeing. For 2020, we defined psychological distress based on the Kessler-6 scale, with six questions assessing non-specific psychological distress in the past four weeks with a sum score of 0-24. While scores <13 indicate no psychological distress, those >12 indicate serious psychological distress.^31^^,^^34^ Total scores for GHQ-5 and Kessler-6 were separately dichotomized to indicate individuals without symptoms of anxiety and depression and those with symptoms of anxiety and depression. The two binary variables were then collapsed into one binary variable for analysis.

The survey included the short version of the Warwick Edinburgh Mental Well-Being Scale (WEMWBS) which measures mental wellbeing. This version includes seven questions on mental wellbeing in the prior two weeks with total scores ranging between 7 and 35 (higher scores indicating better wellbeing). We used the single continuous summary score in the analysis.

Other indicators of mental health included suicidal ideation (“Have you ever been in a situation where you seriously considered taking your own life?”) and attempted suicide (“Have you ever attempted to take your own life?”). Both indicators were analysed as binary variables (No vs. Yes).

### Health-related behaviours

#### Risk alcohol consumption

This was defined using information from the Alcohol Use Disorder Identification Test (AUDIT-C). We used a cut-off value for risk consumption with a score of 5 for women and 6 for men, as recommended by The Public Health Agency of Sweden.^31^

#### Risk gambling

This was defined by using the short version of the Problem Gambling Severity Index (PGSI). We used the same cut-off as recommended by The Public Health Agency of Sweden.^31^ Respondents were categorized as non-gamblers/non-risk gamblers vs. risk gamblers.

#### Substance use

This was defined by two questions (“Have you ever used cannabis, e.g., hashish or marijuana?”) and “Have you ever used an illicit drug other than cannabis (e.g., amphetamine, cocaine, heroin, ecstasy or LSD)?”) about illicit drug use at any time in life. Responses were categorized as no substance use vs. any substance use.

#### Exposure to violence

The survey asked respondents about experiences of specific types of violence such as physical violence, threats, and discrimination in the previous year (for e.g., ‘*In the past 12 months, have you been subjected to physical violence?’)*. Responses were dichotomized into no exposure to violence vs yes.

#### Potential confounders

These included age from the survey, sex at birth from the Total Population register and educational level from the register of education (UREG) categorized as High/Medium/Low/Not applicable. The category ‘not applicable’ included those participants <25 years who have not yet obtained their highest educational levels.

### Analyses

We calculated proportions of sociodemographic characteristics, health, health-related behaviours and exposure to violence, threats, and discrimination according to dual ethnic- and sexual/gender identities. Associations between ethnic- and sexual minority status and all outcomes were estimated using multiple logistic regression (except for mental wellbeing examined using linear regression). Regression models were run with the assumption that ethnicity and sexual identities precede outcomes of interest. All regression models were adjusted for sex at birth, age and educational level. Logistic regression models produce the odds ratio (OR) which is an approximation of risk ratios (RR).^35^ Results were considered statistically significant if the 95% CI did not include 1 (or zero), as appropriate. The likelihood of the regression models being overfitted is substantially reduced due to the large and random sample size.^36^

### Sensitivity analysis

We ran a series of sensitivity analysis using the two alternative sexuality and ethnicity exposure variables described above. First, we calculated distributions of health, health-related behaviours and exposure to violence, threats, and discrimination using the more detailed exposure variables. We also ran all regression models for all outcomes using the alternative exposure variables to examine differences between 1. sexual identity sub-groups (bisexuals, homosexuals and heterosexuals) and 2. cisgender and transgender individuals. These models were run with adjustment for confounders as described above. Results of sensitivity analyses are presented in supplementary files.

All analyses were conducted using population weights calculated by Statistics Sweden to address survey design and to adjust for non-response and were conducted using SPSS (version 27). The calibrated weights are not the same as the commonly used population weights that adjust for response probability. They are a product of a design weight, a non-response weight and multiplied by an adjustment factor. The adjustment factor accounts for auxiliary sociodemographic information of the national population like sex, age, place of birth, civil status, income, residential area, employment status, level of education, profession, social welfare, and sickness benefit as held in the national official registers maintained by Statistics Sweden. This calibration assumes that the selected frame represents the population accurately.^37^

#### Data statement

Data used in this study cannot be shared. However, data from the public health survey can be accessed with the necessary permissions from the Swedish Public Health Agency.

#### Ethics

The Swedish National Public Health Survey has ethics approval from the Swedish National Board of Health and Welfare (Dnr 920031208). This study was further approved by the National Ethics Review Board (Dnr 2020-02847). All participants consented to take part in this survey, and by giving consent also agreeing to research using data collected via the survey.

#### Role of the funding source

The study received no external funding. Linköping University provided funding to access the data from the Public Health Agency of Sweden but had no other role. FF and EM accessed the raw data from the Public Health Agency of Sweden.

## Results

All SGM groups reported higher proportions for mental ill-health compared to their heterosexual peers (for e.g., 35.8% of White-SGM 16·7% of migrant-SGM and 24·7% of refugee-SGM individuals reported suicidal ideation compared to 9·9% of White heterosexuals, Table 1). Regardless of sexuality, EM (migrants and refugees) individuals reported higher proportions of suicidal ideation and attempts compared to White- and Swedish- or Western born heterosexuals. All SGM groups reported higher levels of adverse health-related behaviours (risk alcohol use, risk gambling, and substance use) compared to their heterosexual peers. In general, migrant and refugee groups were less likely to report health-related behaviours (risk alcohol use and substance use) with the exception of risk gambling. Despite the lower levels of adverse health-related behaviours in EM groups, SGM migrant and refugee individuals reported higher levels of risk alcohol consumption and substance use compared to EM heterosexuals (for e.g., 5·3% of migrant-SGM and 4·8% of refugee-SGM individuals reported substance use compared to 1·7% in EM-heterosexuals, Table 1).

### Health and health-related behaviours

**Figure 1:**
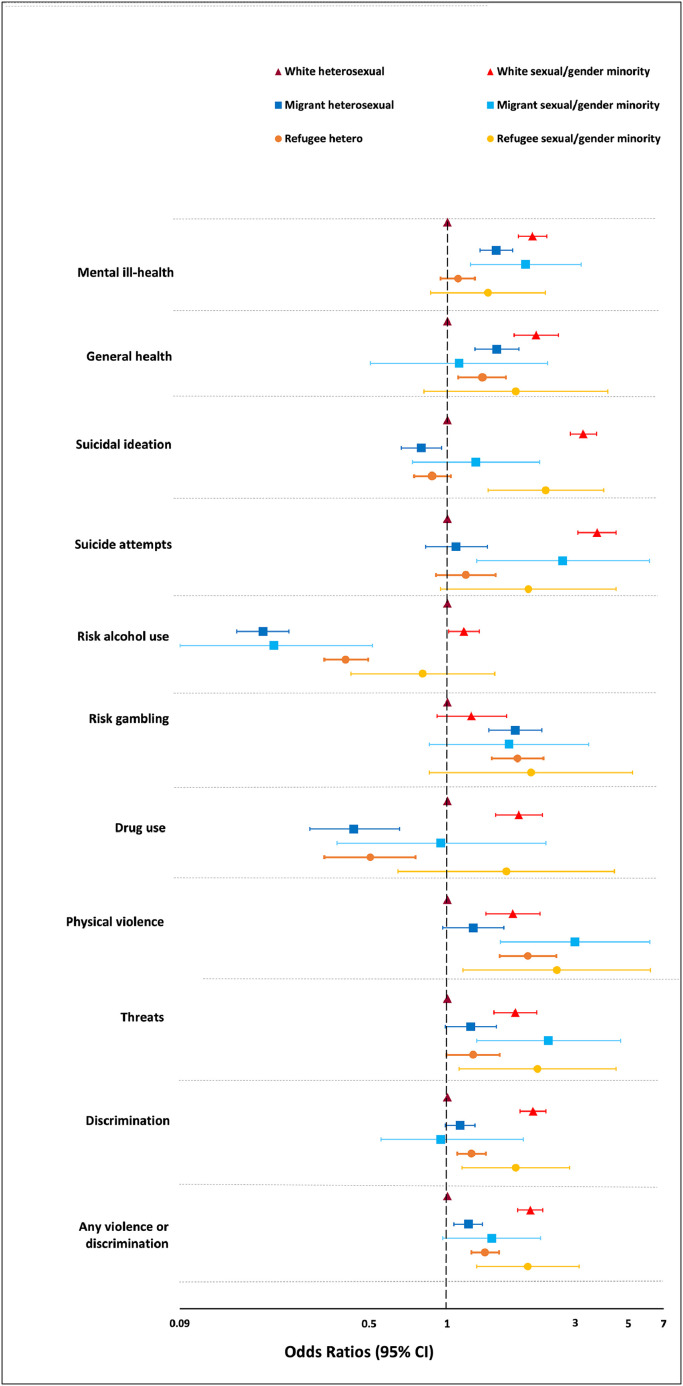
**Odds ratios for health and health-related behaviours based on dual sexual/gender and ethnic (migrant and refugee) identities in 168 952 individuals aged 16-84 years who answered the Swedish National Public Health Survey in 2018-20**. Note: Estimates are from multivariable logistic regression models with adjustment for sex, age and educational level.

Tables 2 and 3, and Figure 1 display results from regression models. Both White-SGM and EM-SGM had higher odds ratios for poorer general health compared to White-heterosexuals, with the highest odds ratios in the White-SGM individuals. White-SGM (OR 2·15, 95% CI: 1·89-2·44), migrant-heterosexuals (OR1·55, 1·34-1·80) and migrant-SGM (OR2·02, 1·23-3·33) had higher odds ratios for mental ill-health compared to White-heterosexuals. We observed similar results in refugee-heterosexuals (OR1·10 0·94-1·28) and refugee-SGM (OR1·44, 0·86-2·41).

**Table 2:** Associations between sexual/gender and ethnic (including migrant and refugee) identities and binary health and well-being in 157,414 individuals aged 16-84 years from the Swedish National Public Health Survey (2018 and 2020). Estimates are from multiple logistic and linear regression models (models adjusted for sex, age, and educational level).

	General health	Mental wellbeing (WEMWBS)^1^	Mental ill-health^2^	Sucidal ideation (lifetime)	Suicide attempts	Risk alcohol use	Risk gambling	Drug use
	OR (95% CI)	β (95% CI)	OR (95% CI)	OR (95% CI)	OR (95% CI)	OR (95% CI)	OR (95% CI)	OR (95% CI)
**Number of yes**	7722	N/A	15118	14185	3803	22347	4254	2487
**White heterosexual**	Ref	Ref	Ref	Ref	Ref	Ref	Ref	Ref
**Number of yes**	415	**N/A**	1162	**1668**	654	1037	171	373
**White Sexual/gender minority**	**2·22 (1·82–2·71)**	**-1·66 (-1·93–-1·38)**	**2·15 (1·89–2·44)**	**3·39 (3·02–3·82)**	**3·84 (3·23–4·55)**	**1·16 (1·01–1·33)**	1·24 (0·91–1·70)	**1·90 (1·54–2·35)**
**Number of yes**	370	**N/A**	**789**	461	187	184	283	71
**Migrant heterosexual**	**1·56 (1·28–1·90)**	**-0·42 (-0·69–-0·14)**	**1·55 (1·34–1·80)**	**0·79 (0·66–0·95)**	1·08 (0·82–1·43)	**0·19 (0·15–0·24)**	**1·84 (1·45–2·34)**	**0·43 (0·29–0·65)**
**Number of yes**	20	N/A	**66**	47	26	17	27	15
**Migrant Sexual /gender minority**	1·11 (0.50–2.46)	-0·02 (-0·98–0·95)	**2·02 (1·23–3·33)**	1·29 (0·73–2·29)	**2·82 (1·30–6·15)**	**0·21 (0·09–0·51)**	1·74 (0·85–3·56)	0·94 (0·37–2·42)
**Number of yes**	278	N/A	570	475	178	315	246	71
**Refugee heterosexual**	**1·37 (1·10–1·69)**	-0·08 (-0·30**–**0·15)	1·10 (0·94–1·28)	0·87 (0·74–1·03)	1·18 (0·90–1·54)	**0·40 (0·33–0·49)**	**1·88 (1·49–2·37)**	**0.50 (0·33–0·75)**
**Number of yes**	16	N/A	53	**62**	30	31	13	12
**Refugee Sexual/gender minority**	1·85 (0·81–4·23)	-0·08 (-0·99**–**0·82)	1·44 (0·86–2·41)	**2·42 (1·44–4·08)**	2·07 (0·94–4·55)	0·80 (0·42–1·53)	2·12 (0·85–5·29)	1·70 (0·64–4·49)

Text in bold indicates estimates with 95% CI that do not include 1 ^2^ Short version of the Warwick Edinburgh Mental Well-Being Scale (WEMWBS) ^3^Assessed using the Kessler-6 and GHQ-5 instruments.

**Table 3:** Associations between sexual/gender and ethnic (including migrant and refugee) identities and experiences of violence in 157,414 individuals aged 16-84 from the Swedish National Public Health Survey. Estimates are from multivariable logistics regression models (models adjusted for sex, age, and educational level).

	Physical violence	Threats	Discrimination	Any violence or discrimination
	OR (95% CI)	OR (95% CI)	OR (95% CI)	OR (95% CI)
**Number of yes**	3391	5306	25518	29076
**White heterosexual**	Ref	Ref	Ref	Ref
**Number of yes**	274	447	1820	1998
**White Sexual/gender minority**	**1·80 (1·41–2·30)**	**1·84 (1·52–2·23)**	**2·16 (1·92–2·42)**	**2·11 (1·88–2·36)**
**Number of yes**	164	272	983	1171
**Migrant heterosexual**	1·26 (0·96-1·66)	1·23 (0·98–1·55)	1·12 (0·98–1·28)	**1·21 (1·06–1·37)**
**Number of yes**	26	30	52	85
**Migrant Sexual/gender minority**	**3·15 (1·61–6·17)**	**2·48 (1·30-4·75)**	0·94 (0·55–1·58)	1·49 (0·96–2·31)
**Number of yes**	174	220	1008	1178
**Refugee heterosexual**	**2·06 (1·60–2·66)**	1·26 (0·99–1·60)	**1·24 (1·09–1·41)**	**1·41 (1·24–1·59)**
**Number of yes**	15	19	80	89
**Refugee Sexual/gender minority**	**2·68 (1·15–6·21)**	**2·25 (1·11-4·55)**	**1·85 (1·14-3·00)**	**2·06 (1·30–3·27)**

Text in bold indicates estimates with 95% CI that do not include 1.

White-SGM (OR3·39, 3·02-3·82) and refugee-SGM (OR2·42, 1·44-4·08) groups had higher odds ratios for suicidal thoughts compared to White-heterosexuals. All three SGM groups [White, (OR3·84, 3·23–4·55), migrant, (OR2·82, 1·30–6·15), and refugee, (OR2·07, 0·95–4·55)] had substantially higher odds for suicide attempts compared to White-heterosexuals.

In general, ethnic minority (regardless of sexual identity) groups were less likely to report risk alcohol and drug use compared to their White peers. For example, risk alcohol consumption was significantly lower in migrant-heterosexuals (OR0·19,0·15-0·24), migrant-sexual minority (OR0·21,0·09-0·51), and refugee-heterosexuals (OR0·40,0·33-0·49). However, migrants and refugees (heterosexual and SGM) were more likely to report risk gambling.

### Experience of different types of violence and discrimination

Compared to White-heterosexual individuals, all other groups had higher odds for experiences of any form of violence or discrimination (for e.g., OR2·11, 1·88-2·36 for White-SGM and OR2·06, 1·30-3·27 for refugee-SGM, Table 3 and Figure 1). Further, White-SGM and refugee-SGM had consistently and significantly higher odds for each form of violence examined separately (physical, threats and discrimination). Migrant-SGM had higher odds for physical violence (OR3·15, 1·61-6·17) and threats (OR2·48, 1·30-4·75) but not discrimination. Migrant- and refugee-heterosexuals also had higher odds for experiences of violence. While migrant-heterosexuals had higher odds for any violence or discrimination (OR1·21, 1·06-1·37), refugee-heterosexuals had higher odds for physical violence (OR2·06, 1·60-2·66) and discrimination (OR1·24, 1·09-1·41).

### Sensitivity analysis

Supplementary Tables 4 and 5 display the distributions of all outcomes according to gender and the more detailed sexual identities (heterosexual, homosexual and bisexual identities), respectively. Compared to White cisgender individuals, all other transgender groups had significantly higher percentages of individuals reporting worse general health, mental ill-health (including suicidal ideation and attempts) and exposure to violence and discrimination. In general, greater percentages of bisexual individuals reported mental ill-health compared to their heterosexual and homosexual peers (with the highest percentages observed in the White bisexual group). While all transgender groups had increased odds for risk gambling, the highest odds were observed for refugee transgender individuals (White: OR1·48, 0·66–3·31, migrant: OR1·42, 0·72-2·82 and refugee OR8·62, 1·94-38·40). Transgender individuals also had higher odds for exposure to physical violence compared to cisgender peers (White transgender: OR3·16 (1·86–5·36), transgender migrants: OR6·31, 2·75-14·52 and transgender refugees OR7·46, 2·97-18·70) (Supplemental Table 7).

When examining differences in associations between SGM subgroups, bisexual individuals had the highest odds for mental ill-health and poorer general health [for example OR2·32 and OR1·86 for mental ill-health and OR2·49 and OR1·89 for general health in White-bisexuals and White-homosexuals, respectively, and compared to White-heterosexuals individuals (Supplemental Table 8)]. For migrant and refugee bisexuals vs. homosexuals, the results were mixed. For health and health-related behaviours, migrant homosexuals had higher odds ratios than bisexuals. For example, migrant homosexuals (OR3·25, 0·89-11·83) had higher odds ratios than migrant bisexuals (OR0·36, 0·11-1·22) for drug use. For refugees, we found the opposite. Refugee homosexuals (OR1·39, 0·60-3·23) had lower odds ratios than refugee bisexuals (OR3·82, 1·90-7·68) for suicidal ideation.

## Discussion

This study found: 1) White-SGM individuals had consistently higher odds for mental ill-health, poorer wellbeing and general health compared to White heterosexual peers. However, findings were not consistent for EM-SGM individuals. 2) There was a marked difference in the observed pattern for health-related behaviours between White- and EM-SGM groups. 3), Both White and EM transgender individuals had higher odds for exposure to physical violence. White Transgender individuals had higher odds for all adverse health outcomes and all types of violence but not for adverse health-related behaviours. Refugee transgender individuals had higher odds for risk gambling. 4) White bisexuals had higher odds for poorer general health, mental ill-health, and drug use compared to White homosexuals. 5). In general, there were no consistent differences in health and health-related behaviour between migrant and refugee bisexuals and homosexuals

To our knowledge, this is the first study to separately examine health and health-related behaviours in SGM migrant and refugee individuals including transgender individuals in a national population-based sample.

We corroborated earlier research showing that SGM individuals have worse mental and general health compared to heterosexuals both internationally and in Sweden.^13^^,^^38^ It also confirms results from the only study on SM-migrants to Sweden which found that being Nordic-born and SM is a stronger risk factor for mental ill-health including suicidal ideation and attempts than being a migrant and SM. However, this study did not include refugees and asylum seekers, only examined mental health, with a smaller sample that was drawn from a regional cohort.^39^ The only other Swedish study to examine mental health in foreign-born individuals residing in Sweden included a sample of 247 SM men, found that country-of-origin stigma was associated with poorer mental health.^40^ This study did not have a heterosexual comparator group, nor did it distinguish between migrants and refugees.

Existing research shows both better^7^ and worse health among EM individuals.^41^ Our findings indicate the complexity of these associations; health, health-related behaviours, and exposure to violence in EM individuals potentially depend on their status as migrants or refugees further compounded by their sexual and gender identities. For example, migrants and refugees were less likely to report risk behaviours (alcohol and drug use) but mental and general health differed by their sexual identity. Refugees as a group have higher risks for poorer health compared to the host population.^8^ However, our results show that while heterosexual refugees reported worse general health and risk gambling, refugee SGM individuals reported higher odds for suicidality. Importantly, regardless of sexuality, being a refugee is significantly associated with exposure to physical violence and discrimination (also seen in all SGM groups in this study).

Previous studies on refugee SGM individuals showed high rates of mental ill-health and exposure to violence.^24^ Our study partially confirms these results (higher odds for suicidal ideation and all types of violence). Unexpectedly we did not find evidence for worse general and mental health. We also corroborate earlier findings that White transgender individuals have poorer health compared to their cisgender peers.^42^ Few studies investigated refugee and migrant transgender health, but the existing research reports high rates of mental ill-health and experiences of violence but findings are based on smaller non-probability samples.^43^ In this study, we found that transgender refugees have significantly poorer general health, not reported before. A previous study found White transgender youth have a higher risk for problem gambling compared to cisgender youth.^44^ Our study is the first to report higher odds of problem gambling in refugee transgender individuals. Lastly, our results are in line with previous Swedish studies that showed higher odds for risk alcohol-use and substance use in sexual minority individuals.^13^

This study benefits from a nationally representative sample with low missing data, and results should be generalizable to the Swedish adult population. The large sample size enabled us to separately examine health in refugee and migrant SM individuals and in sub-groups like bisexual and transgender individuals. Further, the proportions of ethnic and SM individuals who took part in the health survey are similar to the proportions of these groups in the general Swedish population.^45^

Self-reported ethnicity is the ‘gold-standard’ for assessing ethnicity.^46^ Nonetheless, country of birth is considered a reasonable and often used substitute in the absence of self-identified ethnicity and is widely used in epidemiological studies in Europe.^47^, ^48^, ^49^ However, we recognize that using country of birth as a proxy has certain limitations; individuals born in Sweden are categorised as Swedish, even though they may identify as EM. Further, distinct EM groups such as the Kurdish cannot be identified by country of birth. We acknowledge the Swedish- and Western-born group includes individuals who do not identify as White but we nonetheless expect the overwhelming majority in this category to be of White/ European-origin. We had to combine asylum seekers, persons in need of subsidiary protection and refugees in the same group as it is impossible to distinguish these categories of individuals in the dataset. Nonetheless, the distinction between these groups is not always clear and they are often used interchangeably.^50^^,^^51^ Lastly, the framework (developed in collaboration with the Swedish Migration Agency) to ascertain and distinguish migrant and refugee individuals was robust and used key variables like birth country, migration year, refugee status in high quality national registers alongside historical trends in national migration policy.

Despite the large sample size and population-based design, we had relatively small numbers of individuals who identified as being both EM and SGM, with particularly smaller numbers in the refugee-SM and migrant/refugee-transgender groups. This results in loss of statistical power and affects the precision of some observed estimates (for e.g., the wide confidence intervals related to estimates for transgender individuals). While these estimates would be considered ‘statistically significant’, we advise caution in interpreting the findings. However, our findings indicate overall higher risk for adverse health and experiences in minority groups and highlights the need for larger studies with adequate statistical power. The small number of individuals with dual minority identities also prevented analysis of differences between female and male sexual minorities (Supplementary Table 3) and examining potential non-linear relationships in some outcomes of interest which could have ≥2 categories. We used two different measures for mental health; Kessler-6, and GHQ-5 due to changes in the health survey between 2018 and 2020. Further, while categorization (dichotomizing) of continuous variables like Kessler-6 and GHQ-5 results in loss of statistical power, it facilitates comparison with similar studies as it is common to categorize these variables based on clinically validated cut-offs.^52^ A low response rate, common with national health surveys of this kind is a limitation, but the use of population weights helps compensate for non-response, and the sample remains nationally representative.^31^ Lastly, the 2020 survey did not include any questions related to the covid-19 pandemic. There is a possibility that some participants might have been affected by covid-related issues but unfortunately, we cannot ascertain this impact in our study, which needs to be investigated in future studies.

The substantially higher odds for risk gambling in refugee- SGM individuals is a novel finding. Previous reports highlight that risk gambling is more common among migrants in Sweden.^53^ There has been an increased interest in health inequalities resulting from gambling-related harm, suggesting that some EM groups have worse outcomes from gambling than others.^54^ This is relevant to public health policy as gambling is rarely examined and discussed in studies on refugee/migrant health.

We hypothesized that EM-SGM individuals would have worse health due to the additive effects of two or more minority identities but found limited evidence for this. The few studies that examined health in refugee SGM individuals suggest a cumulative effect of being both a refugee and SGM, but evidence is scarce. Our findings highlight that the problem is likely more complex than a gradient effect and factors other than minority status (for e.g., expectations on equal treatment in society) might affect mental health.

Other explanations include the multiple minority stress model that states stressors associated with being sexual and ethnic minority move through the same pathways.^55^ This model hypothesizes that poorer health outcomes in individuals with one or more minority identities results from multiple and repeated exposures to stigma, discrimination and microaggressions. This may explain the observed higher odds for violence and discrimination found in all minority groups including the White-SM, EM-heterosexual and dual minority groups in this study.

Further, these findings were more pronounced in transgender individuals. However, our results cannot only be explained by the cumulative effects of minority identities. For example, White SGM had worse mental and general health, suicidality, and risk alcohol and drug use compared to dual minority individuals, which contradicts the additive effects of more than one minority identity on health. Earlier studies showed that some pathways leading to mental ill-health in refugees and migrants include social exclusion, isolation, inability to access the labour market, and exposure to discrimination in the host country.^56^ This could potentially explain some of our results as the EM-SGM group are potentially more exposed to a greater burden of discrimination associated with their ethnicity and sexuality leading to poorer health, compared to heterosexual peers. These mechanisms can be appropriately investigated in longitudinal data which is currently unavailable in Sweden.

The intersectionality framework theory is another potential and often cited explanation for adverse health in individuals with multiple minority identities.^27^^,^^28^ This framework theory helps understand how unique and multiple social identities (like ethnicity, gender, sexuality, faith belief etc.) intersect at the individual-level, reflecting various multiple and reciprocal systems of discrimination that impact health in individuals with ≥2 minority identities.^27^^,^^28^ Thus, each individual experiences their own unique forms of discrimination and oppression associated with their identities. These social identities are not isolated from one another or simply additive but are interdependent and mutually constitutive, i.e., they should be examined jointly to better understand the interdependence of minority identities. In Sweden, research has primarily examined health associated with one minority identity (most often ethnicity) and disregarded the potential impact of intersecting multiple minority identities. The intersectionality theory can explain some of our results, including contradictory findings. Minority groups may be differently impacted based on their unique intersecting minority identities and the environments they live in, including the host country's policies. For example, while White-SGM individuals had increased risk for all adverse health-related behaviours except risk gambling, the opposite was observed in EM-SGM individuals. This could reflect differences in coping mechanisms and/or recreational activities intrinsically associated with unique identities. White-SGM individuals are more likely to report excessive alcohol and drug use, often a coping mechanism to deal with discrimination and stress associated with being SGM,^12^^,^^57^ and non-White individuals potentially use other forms of more culturally accepted coping mechanisms like gambling. Lived experiences and their intrinsic associations with unique identities and consequences on health can be further impacted by wider society and the host country's health policies. Currently, Swedish policies on gambling are more general without any specific guidelines related to ethnic and/or sexual identities and our results suggest that this might be relevant in future policies.

Migrant and refugee individuals who may have experienced terrible events in their home countries (for example, discrimination associated with SGM identities or experiences related to war/conflict), might feel relieved and safe after arrival in a host country. This differs from individuals who grow-up and live in a country like Sweden with liberal SGM rights but still experience discrimination, which can lead to disappointment and depression.

We hypothesize that the exposure of interest i.e., ethnic and sexual identities is conceptualized within individuals before the occurrence of outcomes (i.e., temporality). Nonetheless, as this is a cross-sectional study, we do not interpret findings as an indication of causality. This highlights the need for longitudinal data on individuals with self-identified ethnicity and sexuality which currently does not exist in Sweden.

To conclude, we found White SGM individuals reported worse health and were more likely to engage in risk behaviours in Sweden. These associations differed in migrant and refugee SGM groups. Access to care is already difficult for migrant and refugee individuals and SGM minorities.^58^^,^^59^ Barriers include language (inability to speak Swedish), stigma, low awareness and different help-seeking behaviours in refugees^58^ and heteronormative attitudes towards sexual minorities.^59^ There is thus good reason to assume that this access and use of care might be even lower in SGM migrants and refugees. Hence, there is need for more research and practices into increasing access to care for this population as well as developing interventions specifically tailored to meet their needs. To be able to fully investigate these differences in the future more detailed data sets including ethnicity and sexual and gender minorities are warranted. Future public health policy needs to address existing barriers such as improving access to healthcare services and training of professionals including being made aware of difficulties faced by SGM individuals. A specific focus should be on risk gambling. Further, preventive measures to reduce exposure to violence and discrimination in both SGM- and EM-groups. Finally, there is a need to involve SGM groups in informing public policy since their views on these health disparities are often not considered.

## Contributors

EM, AK and FF conceived the idea and designed the study. EM and FF conducted the data collection and EM, FF, LK and AK analyzed the data. EM drafted the manuscript. All authors critically revised the work and approved the final submitted version.

## Data sharing statement

Data used this study cannot be shared. However, data from the public health survey can be accessed with the necessary permissions from the Swedish Public Health Agency.

## Declaration of interests

EM has received funding for accessing the data from Linköping University. Linköping University and Save the Children has also provided salary during the period of the study. EM has also received royalty for a chapter in the book *En förskola för alla* from Liber.

LK has received fees for the ethical application and data acquisition and salaries from the University of Linköping, National competence center Barnafrid. Laura Korhonen has received a grant for the research project “Long way to shelter – longitudinal studies on factors important for early screen and coordinated actions to promote mental health among adolescent and young adult migrants” from The Swedish Research Council for Health, Working Life and Welfare (FORTE), grant number Dnr 2019-12-01 and royalty for a chapter in the book *En förskola för alla* from Liber.

## Appendix Supplementary materials

Image, application 1
