# Supplementary material for: Health and health-related behaviours in refugees and migrants who self-identify as sexual or gender minority – A National population-based study in Sweden
Source: eClinicalMedicine. 2022 Sep 1;52:101641. doi: 10.1016/j.eclinm.2022.101641 (PMC9596319; doi:10.1016/j.eclinm.2022.101641)
Supplement: Supplementary file 1 [file mmc1.docx]

Supplementary Material

# *Mental and general health in refugees and migrants who self-identify as sexual- and/or gender minority – A National population-based Study in Sweden*

**Authors:**

Erica Mattelin, Frida Fröberg, Laura Korhonen, Amal R. Khanolkar.

**Supplementary Table 1: A detailed description of all variables and outcomes as assessed in the Swedish national public health survey ‘Health on equal terms’ (2018-20) and used in this study**

| **Indicator and outcome variables** | **As originally assessed in the national public health survey** | **Options** | **Final form used in analysis** |
| --- | --- | --- | --- |
| Sexual identity | *How would you define your sexual identity?* | 1 heterosexual  2 bisexual  3 homosexual  4 other  5 don’t know | 1 Heterosexual  2-3 Sexual minority  4-5 Excluded if not “yes” on gender identity |
| Gender identity | *Are you or have you been a transgender person?* | 1 yes  2 no  3 don’t know | 1 Sexual minority  3 Excluded if not sexual minority on sexual identity |
| Mental ill-health | For 2020, Kessler-6  *In the past month, how often did you feel*   1. *nervous?* 2. *Hopeless* 3. *Restless or fidgety?* 4. *so depressed that nothing could cheer you up?* 5. *that everything was an effort?* 6. *worthless?* | 4 All the time  3 Most of the time  2 Some of the time  1 A little of the time  0 None of the time | Total score: 0-12 No mental ill-health |
|  |  |  | 13-24 Mental ill-health |
|  | For 2018, General Health Questionnaire (GHQ) -5  *Over the past few weeks:*   1. *have you felt constantly unhappy and depressed?* 2. *have you been losing confidence in yourself?* 3. *have you felt constantly tense?* 4. *have you thought of yourself as a worthless person?* 5. *have you felt that you couldn’t overcome your difficulties?* | 0 Not at all  0 No more than usual  1 Rather more than usual  1 Much more than usual | Score: 2< mental ill-health  2> No mental ill-health |
| Mental wellbeing  (The Short Warwick-Edinburgh Mental Wellbeing Scale - WEMWBS) | *I've been feeling optimistic about the future*  *I've feeling useful*  *I've been feeling relaxed*  *I've been dealing with problems well*  *I've been thinking clearly*  *I've been feeling close to other people*  *I've been able to make up my own mind about things* | 1 All of the time  2 Often  3 Some of the time  4 Rarely  5 None of the time | Total sum score (range 7 to 35) |
| Suicidal ideation | *Have you ever been in a situation where you seriously considered taking your own life* | 1 No  2 Yes, during the past 12 months  3 Yes, more than a year ago | 1 No  2-3 Yes |
| Suicide attempts | *Have you ever attempted to take your own life?”.* | 1 No  2 Yes, during the past 12 months  3 Yes, more than a year ago | 1 No  2-3 Yes |
| General health | *How would you rate your general health?* | 1 Very good  2 Good  3 Moderate  4 Bad  5 Very bad | 1-2 Very good or good  3 Moderate  4-5 Bad or very bad |
| Risk alcohol consumption | Alcohol Use Disorders Identification Test | Total sum score of 0–12 | <5 risk consumption for women |
|  |  |  | <6 risk consumption for men |
| Gambling | Problem gambling severity index (PGSI):  During the past 12 months, have you:  a. gambled with more money than you really could afford to lose?  b. needed to gamble with larger amounts to get the same sense of thrill?  c. returned another day to win back money that you lost?  d.borrowed money or sold something to get money for gambling? | 0 Never  1 Sometimes  2 Often  3 Almost always | <0risk gambling |
| Substance use | *Have you ever used cannabis (e.g., hashish or marijuana)?* | 1 No  2 Yes, more than 12 months ago  3 Yes, in the past 12 months  4 Yes, in the past 30 days | 1 No  2-4 Yes |
|  | *Have you ever used an illicit drug other than cannabis (e.g. amphetamine, cocaine, heroin, ecstasy or LSD)?* |  |  |
| Physical violence | *In the past 12 months, have you been subjected to physical violence?* | 1 Yes  2 No | Yes  No |
| Threats | *In the past 12 months, have you been subjected to a threat or threats of violence that made you afraid?* | 1 Yes  2 No | Yes  No |
| Discrimination | *In the past three months, have you been treated in a way that made you feel discriminated against?* | 1 No  2 Yes, sometimes  3 Yes, several times | 1 No  2-3 Yes |
| Any exposure | *If yes of any above* |  |  |
| Educational level | Educational level from the register of education | 1 No education or Elementary school, primary school  2 Two years of upper secondary s school or high school or 3–4 years of upper secondary school or high school  3 Some higher education/ University or college, less than 3 years/ University or college, 3 years or more.  4 Participant is below 25 years of age | 1 Low  2 Medium  3 High  4 Education level is not applicable. |

**Supplementary Table 2: Strategy used to identify ethnic minority individuals (migrant and refugee identities) who answered the National Public Health Survey in 2018 and 2020. The strategy outlined was developed in consultation with Swedish Board of Migration (*Migrationsverket*)**

| Participants were categorized as refugees if they met the criteria for A *AND* B | A They were born outside of Sweden (Country of birth from The Total Population Register) |
| --- | --- |
|  | B Migrated during 1939-1945 regardless of country (Year of Immigration – The Total Population Register) OR Has been categorized as a refugee according to the Swedish system (Categorized as refugee (FlyKat)– STATIV) OR Immigrated from Former Yugoslavia between 1980 - Onwards (Country of birth+ InvAr – The Total Population Register) OR Immigrated from Iran before 2007 (Country of birth + Year of Immigration– The Total Population Register) OR Immigrated from Syria, Eritrea, Afghanistan, Somalia (Country of birth– The Total Population Register) OR Immigrated from the Soviet Union until 2010 (Country of birth – The Total Population Register) OR Immigrated from the Iraq until 2010 (Country of birth – The Total Population Register) |
| Participants were categorized as migrants if they met the criteria for C *AND* D | C They were born outside of Sweden (Country of Birth - The Total Population Register) |
|  | D Immigrated from Former Yugoslavia before 1980 (Country of birth + Year of Immigration – The Total Population Register) OR Immigrated from Iran after 2007 (Country of birth + Year of Immigration– The Total Population Register) OR Migrated from South America, Asia, Africa (Country of birth – The Total Population Register) OR Europe but not EU after 1945 (Country of birth – The Total Population Register) OR Immigrated from the Iraq after 2010 (Country of birth – The Total Population Register) OR Immigrated from the Soviet Union after 2010 (Country of birth – The Total Population Register) |
| Participants were categorized as Swedish- or Western-born (White) if they met the criteria for E *OR*  F | E They were born in Sweden OR Oceania OR North America (Country of birth -  The Total Population Register) |
|  | F They were born in the Nordic Countries OR EU28 after 1945 (Year of Birth – Year of Immigration – The Total Population Register). |

**Supplementary Table 3. Descriptive characteristics by ethnic and detailed sexual identities, and sex at birth of 157,414 individuals who answered the Swedish National Public Health Survey in 2018 and 2020. The numbers are unweighted counts (n) and unweighted percentages (%)**

| Females | | | | | | | | | | | | | | | | | | | |
| --- | --- | --- | --- | --- | --- | --- | --- | --- | --- | --- | --- | --- | --- | --- | --- | --- | --- | --- | --- |
| Outcomes of interest | White-heterosexual | | White-bisexual | | White-homosexual | | Migrant heterosexual | | Migrant bisexual | | Migrant homosexual | | Refugee heterosexual | | Refugee bisexual | | Refugee homosexual | |  |
|  | N | % | N | % | N | % | N | % | N | % | N | % | N | % | N | % | N | % |  |
| Physical violence | 1660 | 2·2 | 135 | 6·4 | 25 | 4·5 | 74 | 3·7 | 5 | 6·8 | 2 | 12·5 | 96 | 3·9 | 2 | 2·7 | 3 | 6·7 |  |
| Threats | 3081 | 4·0 | 235 | 11·1 | 45 | 8·2 | 134 | 6·7 | 6 | 8·2 | 0 | 0·0 | 122 | 4·9 | 6 | 8·0 | 3 | 6·7 |  |
| Discrimination | 16848 | 21·9 | 1077 | 50·7 | 212 | 38·1 | 494 | 24·7 | 21 | 29·2 | 2 | 12·5 | 631 | 25·5 | 34 | 45·3 | 13 | 28·3 |  |
| Any of above | 18480 | 23·9 | 1135 | 53·3 | 229 | 41·1 | 574 | 28·6 | 26 | 35·6 | 3 | 18·8 | 711 | 28·5 | 35 | 46·7 | 16 | 34·8 |  |
| Bad/very bad general health | 4403 | 5·7 | 219 | 10·3 | 43 | 7·7 | 213 | 10·7 | 4 | 5·5 | 1 | 6·3 | 181 | 7·3 | 5 | 6·6 | 4 | 8·7 |  |
| Mental ill-health | 9508 | 12··3 | 677 | 31·9 | 118 | 21·2 | 409 | 20·4 | 21 | 28·8 | 4 | 25·0 | 370 | 14·9 | 12 | 22·4 | 8 | 17·4 |  |
| Suicidal ideation | 8507 | 11·1 | 993 | 46·9 | 163 | 29·4 | 238 | 11·9 | 14 | 20·0 | 4 | 25·0 | 307 | 12·4 | 26 | 34·7 | 9 | 20·0 |  |
| Suicide attempts | 2466 | 3·2 | 411 | 19·4 | 61 | 11·1 | 103 | 5·2 | 7 | 9·7 | 4 | 25·0 | 116 | 4·7 | 10 | 13·2 | 5 | 11·1 |  |
| Risky alcohol use | 9759 | 12·6 | 476 | 22·4 | 107 | 19·2 | 47 | 2·3 | 4 | 5·5 | 1 | 6·3 | 142 | 5·7 | 8 | 10·5 | 8 | 17·4 |  |
| Substance use^2^ | 816 | 1·1 | 206 | 9·7 | 20 | 3·6 | 19 | 0·9 | 3 | 4·1 | 1 | 6·3 | 35 | 1·4 | 5 | 6·6 | 0 | 0·0 |  |
| Risk gambling | 1368 | 1·8 | 50 | 2·4 | 16 | 2·9 | 56 | 2·8 | 1 | 1·4 | 3 | 18·8 | 93 | 3·8 | 3 | 4·1 | 3 | 6·5 |  |
| Males | | | | | | | | | | | | | | | | | | | |
| Outcomes of interest | White-heterosexual | | White-bisexual | | White-homosexual | | Migrant heterosexual | | Migrant bisexual | | Migrant homosexual | | Refugee heterosexual | | Refugee bisexual | | Refugee homosexual | |  |
|  | N | % | N | % | N | % | N | % | N | % | N | % | N | % | N | % | N | % |  |
| Physical violence | 1764 | 2·6 | 53 | 6·3 | 28 | 3·8 | 98 | 4·2 | 8 | 8·4 | 3 | 6·7 | 83 | 4·2 | 2 | 4·3 | 3 | 5·8 |  |
| Threats | 2265 | 3·4 | 69 | 8·3 | 58 | 8·0 | 146 | 6·3 | 9 | 9·9 | 7 | 16·3 | 100 | 5·8 | 3 | 6·4 | 5 | 9·6 |  |
| Discrimination | 8741 | 13·2 | 244 | 29·3 | 216 | 29·8 | 498 | 21·4 | 12 | 12·9 | 8 | 17·8 | 383 | 22·3 | 10 | 21·3 | 17 | 32·7 |  |
| Any of above | 10710 | 16·1 | 280 | 33·5 | 240 | 33·1 | 616 | 26·4 | 24 | 25·5 | 13 | 28·9 | 476 | 27·7 | 11 | 23·4 | 18 | 34·6 |  |
| Bad/very bad general health | 3353 | 5·1 | 72 | 8·7 | 47 | 6·4 | 160 | 6·9 | 7 | 7·4 | 5 | 11·4 | 98 | 5·7 | 4 | 8·7 | 2 | 3·8 |  |
| Mental ill-health | 5667 | 8·5 | 183 | 21·8 | 127 | 17·4 | 393 | 16·9 | 17 | 18·3 | 11 | 24·4 | 204 | 11·9 | 13 | 27·7 | 11 | 21·2 |  |
| Suicidal ideation | 5728 | 8·7 | 283 | 33·9 | 177 | 24·3 | 227 | 9·8 | 15 | 16·0 | 10 | 22·2 | 172 | 10·0 | 13 | 27·7 | 10 | 19·2 |  |
| Suicide attempts | 1353 | 2·0 | 103 | 12·4 | 63 | 8·7 | 90 | 3·9 | 6 | 6·5 | 3 | 6·7 | 66 | 3·8 | 6 | 12·8 | 5 | 9·6 |  |
| Risky alcohol use | 12652 | 19·0 | 209 | 25·0 | 181 | 24·8 | 138 | 5·9 | 8 | 8·4 | 3 | 6·7 | 177 | 10·3 | 3 | 6·5 | 8 | 15·4 |  |
| Substance use^2^ | 1675 | 2·5 | 97 | 11·6 | 46 | 6·3 | 53 | 2·3 | 2 | 2·1 | 8 | 17·8 | 37 | 2·1 | 3 | 6·4 | 3 | 5·8 |  |
| Risk gambling | 2906 | 4·4 | 47 | 5·6 | 36 | 5·0 | 229 | 9·9 | 16 | 17·0 | 5 | 11·4 | 155 | 9·1 | 1 | 2·1 | 4 | 7·7 |  |

**Supplementary table 4. Descriptive characteristics by ethnic and gender identities of 164,118 individuals who answered the Swedish National Public Health Survey in 2018 and 2020. The numbers are unweighted counts (n) and unweighted percentages (%)**

|  | **White cisgender**  **(N=152827)** | | **White transgender**  **(N=693)** | | **Migrant cisgender**  **(N=5442)** | | **Migrant transgender**  **(N=109)** | | | **Refugee cisgender**  **(N=4991)** | | **Refugee transgender**  **(N=56)** | | **Test for difference^1^** |  |
| --- | --- | --- | --- | --- | --- | --- | --- | --- | --- | --- | --- | --- | --- | --- | --- |
| Outcomes | N | % | N | % | N | % | N | % | | N | % | N | % |  |  |
| **General health** Good to moderate | 142818 | 94·4 | 605 | 88·5 | 4885 | 90·5 | 98 | 89·9 | | 4587 | 92·7 | 49 | 90·7 | <0·001 |  |
| Bad or very bad | 8532 | 5·6 | 79 | 11·5 | 512 | 9·5 | 11 | 10·1 | | 363 | 7·3 | 5 | 9·3 |  |  |
| **Mental well-being^2^** Median  (25^th^/75^th^ percentile) | 28 | 26/31 | 28 | 24/30 | 28 | 25/31 | 29 | 26/33 | 28 | | 25/31 | 28 | 25/32 |  |  |
| **Mental health** Mental ill-health^3^ | 16824 | 11·1 | 165 | 23·8 | 1038 | 19·2 | 22 | 20·8 | | 733 | 14·8 | 8 | 14·5 | <0·001 |  |
| No | 135083 | 88·9 | 527 | 76·2 | 4357 | 80·8 | 84 | 79·2 | | 4212 | 85·2 | 47 | 85·5 |  |  |
| **Suicidal ideation**  Yes, ever | 16286 | 9·7 | 198 | 28·9 | 596 | 11·1 | 15 | 14·0 | | 572 | 11·6 | 14 | 25·5 | <0·001 |  |
| No | 135472 | 89·3 | 488 | 71·1 | 4779 | 88·9 | 92 | 86·0 | | 4374 | 88·4 | 41 | 74·5 |  |  |
| **Suicide attempts**  Yes, ever | 4624 | 3·0 | 80 | 11·7 | 257 | 4·8 | 16 | 14·8 | | 233 | 4·7 | 12 | 21·8 | <0·001 |  |
| No | 147147 | 97·0 | 605 | 88·3 | 5121 | 95·2 | 92 | 85·2 | | 4711 | 95·3 | 43 | 78·2 | <0·001 |  |
| **Health-related behaviors in the previous 12 months** |  |  |  |  |  |  |  |  | |  |  |  |  |  |  |
| **Risk gambling^4^** |  | | | | | | | | | | | | |  |  |
| Yes | 4751 | 3·1 | 28 | 4·1 | 394 | 7·3 | 19 | 17·9 | | 329 | 6·7 | 6 | 10·9 | 0·005 |  |
| No | 146490 | 96·9 | 652 | 95·9 | 4975 | 92·7 | 87 | 82·1 | | 4611 | 93·3 | 49 | 89·1 |  |  |
| **Substance use^5^** |  | | | | | | | | | | | | |  |  |
| Yes | 2930 | 1·9 | 24 | 3·5 | 87 | 1·6 | 1 | 0·9 | | 87 | 1·7 | 3 | 5·6 | <0·001 |  |
| No | 149338 | 98·1 | 664 | 96·5 | 5331 | 98·4 | 107 | 99·1 | | 4889 | 98·3 | 51 | 94·4 |  |  |
| **Risk alcohol use** Yes | 23856 | 15·5 | 105 | 15·2 | 240 | 4·4 | 6 | 5·6 | | 374 | 7·5 | 8 | 14·3 | <0·001 |  |
| No | 128812 | 84·5 | 586 | 84·8 | 5180 | 95·6 | 102 | 94·4 | | 4607 | 92·5 | 48 | 85·7 |  |  |
| **Exposure to any kind of violence or discrimination** Yes | 31714 | 20·8 | 266 | 38·7 | 1429 | 26·3 | 39 | 35·8 | | 1390 | 28·0 | 19 | 34·5 | <0·001 |  |
| No | 120504 | 79·2 | 421 | 61·3 | 3998 | 73·7 | 70 | 64·2 | | 3583 | 72·0 | 36 | 65·5 |  |  |
| **Exposure to threats**  Yes | 5862 | 3·9 | 72 | 10·6 | 358 | 6·7 | 14 | 13·2 | | 288 | 5·8 | 7 | 13·0 | <0·001 |  |
| No | 145456 | 96·1 | 606 | 89·4 | 5020 | 93·3 | 92 | 86·8 | | 4659 | 94·2 | 47 | 87·0 |  |  |
| **Exposure to discrimination**  Yes | 27923 | 18·4 | 215 | 31·5 | 1158 | 21·5 | 21 | 19·8 | | 1188 | 24·0 | 12 | 21·8 | <0·001 |  |
| No | 123853 | 81·6 | 468 | 68·5 | 4235 | 78·5 | 85 | 80·2 | | 3765 | 76·0 | 43 | 78·2 |  |  |
| **Exposure to physical violence**  Yes | 3753 | 2·6 | 57 | 8·3 | 243 | 4·5 | 19 | 17·4 | | 212 | 4·3 | 10 | 18·2 | <0·001 |  |
| No | 148158 | 97·4 | 628 | 91·7 | 5157 | 95·5 | 90 | 82·6 | | 4741 | 95·7 | 45 | 81·8 |  |  |

^1^p values are for a test of means or equal proportions^. 2^ Short version of the Warwick Edinburgh Mental Well-Being Scale (WEMWBS) ^3^ Mental health based on the General Health Questionnaire (GHQ) used in 2018 and the Kessler-6 used in 2020. ^4^Score of more than one on Problem Gambling Severity Index.  ^5^Any use of drugs during the last year.

**Supplementary table 5. Descriptive characteristics by ethnic and detailed sexual identities of 157,414 individuals who took part in the Swedish National Public Health Survey in 2018 and 2020. The numbers are unweighted counts (n) and unweighted percentages (%)**

| Outcomes of interest | White-heterosexual | | White-bisexual | | White-homosexual | | Migrant heterosexual | | | Migrant bisexual | | | | Migrant homosexual | | | | Refugee heterosexual | | | | Refugee bisexual | | | | Refugee homosexual | | | | Test for difference |
| --- | --- | --- | --- | --- | --- | --- | --- | --- | --- | --- | --- | --- | --- | --- | --- | --- | --- | --- | --- | --- | --- | --- | --- | --- | --- | --- | --- | --- | --- | --- |
|  | N | % | N | % | N | % | N | % | | N | | % | | N | | % | | N | | % | | N | | % | | N | | % | |  |
| **General health** Good to moderate | 135001 | 94·6 | 2658 | 90·1 | 1196 | 93·0 | 3949 | 91·4 | | 156 | | 93·4 | | 54 | | 90·0 | | 3916 | | 93·3 | | 113 | | 92·6 | | 92 | | 93·9 | | <0·001 |
| Bad or very bad | 7756 | 5·4 | 291 | 9·9 | 90 | 7·0 | 373 | 8·6 | | 11 | | 6·6 | | 6 | | 10·0 | | 279 | | 6·7 | | 9 | | 7·4 | | 6 | | 6·1 | |  |
| **Mental well-being** Median  (25^th^/75^th^ percentile) | 28 | 27/31 | 27 | 23/29 | 28 | 25/30 | 28 | 25/31 | | 28 | | 25/31 | | 27 | | 22/32 | | 28 | | 25/31 | | 28 | | 25/30 | | 38 | | 25/31 | |  |
| **Mental ill-health** Yes | 15175 | 10·6 | 860 | 29·0 | 245 | 19·1 | 802 | 18·5 | | 38 | | 22·9 | | 15 | | 24·6 | | 574 | | 13·7 | | 30 | | 24·4 | | 19 | | 19·4 | | <0·001 |
| No | 128150 | 89·4 | 2102 | 71·0 | 1040 | 80·9 | 3526 | 81·5 | | 128 | | 77·1 | | 46 | | 75·4 | | 3619 | | 86·3 | | 93 | | 75·6 | | 79 | | 80·6 | |  |
| **Suicidal ideation**  Yes, ever | 14235 | 9·9 | 1276 | 43·2 | 340 | 26·5 | 465 | 10·8 | | 29 | | 17·7 | | 14 | | 23·0 | | 479 | | 11·4 | | 39 | | 32·0 | | 19 | | 19·6 | | <0·001 |
| No | 128917 | 90·1 | 1675 | 56·8 | 942 | 73·5 | 3847 | 89·2 | | 135 | | 82·3 | | 47 | | 77·0 | | 3715 | | 88·6 | | 83 | | 68·0 | | 78 | | 80·4 | |  |
| **Suicide attempts**  Yes, ever | 3819 | 2·7 | 514 | 17·4 | 124 | 9·7 | 193 | 4·5 | | 13 | | 7·9 | | 7 | | 11·5 | | 182 | | 4·3 | | 16 | | 13·0 | | 10 | | 10·3 | | <0·001 |
| No | 139348 | 97·3 | 2438 | 82·6 | 1156 | 90·3 | 4121 | 95·5 | | 151 | | 92·1 | | 54 | | 88·5 | | 4008 | | 95·7 | | 107 | | 87·0 | | 87 | | 89·7 | |  |
| **Health-related behaviors in the previous 12 months** |  |  |  |  |  |  |  |  | |  | |  | |  | |  | |  | |  | |  | |  | |  | |  | |  |
| **Risk gambling^3^** Yes | 4274 | 3·0 | 97 | 3·3 | 52 | 4·1 | 285 | 6·6 | | 17 | | 10·2 | | 8 | | 13·3 | | 248 | | 5·9 | | 4 | | 3·3 | | 7 | | 7·1 | | <0·001 |
| No | 138462 | 97·0 | 2852 | 96·7 | 1230 | 95·9 | 4023 | 93·4 | | 149 | | 89·8 | | 52 | | 86·7 | | 3938 | | 94·1 | | 117 | | 96·7 | | 91 | | 92·9 | |  |
| **Risk alcohol use** Yes | 22441 | 15·6 | 685 | 23·1 | 288 | 22·4 | 185 | 4·3 | | 12 | | 7·1 | | 4 | | 6·6 | | 319 | | 7·6 | | 11 | | 9·0 | | 16 | | 16·3 | | <0·001 |
| No | 121446 | 84·4 | 2276 | 76·9 | 1000 | 77·6 | 4155 | 95·7 | | 156 | | 92·9 | | 57 | | 93·4 | | 3902 | | 92·4 | | 111 | | 91·0 | | 82 | | 83·7 | |  |
| **Substance use** Yes | 2491 | 1·7 | 303 | 10·2 | 1218 | 94·9 | 72 | 1·7 | | 5 | | 3 | | 9 | | 14·8 | | 72 | | 1·7 | | 8 | | 6·5 | | 3 | | 3·1 | | <0·001 |
| No | 141118 | 98·3 | 2655 | 89·8 | 66 | 5·1 | 4263 | 98·3 | | 162 | | 97·0 | | 52 | | 85·2 | | 4142 | | 98·3 | | 115 | | 93·5 | | 95 | | 76·9 | |  |
| **Exposure to any kind of violence or discrimination** Yes | 29190 | 20·3 | 1415 | 47·8 | 469 | 36·6 | 1190 | 27·4 | | 50 | | 29·9 | | 16 | | 26·2 | | 1187 | | 28·2 | | 46 | | 37·7 | | 34 | | 34·7 | | <0·001 |
| No | 114393 | 79·7 | 1548 | 52·2 | 814 | 63·4 | 3153 | 72·6 | | 117 | | 70·1 | | 45 | | 73·8 | | 3024 | | 71·8 | | 76 | | 62·3 | | 64 | | 65·3 | |  |
| **Exposure to threats**  Yes | 5346 | 3·7 | 304 | 10·3 | 103 | 8·1 | 280 | 6·5 | | 15 | | 9·1 | | 7 | | 12·1 | | 222 | | 5·3 | | 9 | | 7·4 | | 8 | | 8·2 | | <0·001 |
| No | 137388 | 96·3 | 2642 | 89·7 | 1169 | 91·9 | 4025 | 93·5 | | 149 | | 90·9 | | 50 | | 87·9 | | 3967 | | 94·7 | | 113 | | 92·6 | | 89 | | 91·8 | |  |
| **Exposure to discrimination** Yes | 25589 | 17·9 | 1321 | 44·7 | 428 | 33·4 | 992 | 23·0 | | 33 | | 20·0 | | 10 | | 16·4 | | 1014 | | 24·2 | | 44 | | 36·1 | | 30 | | 30·6 | | <0·001 |
| No | 117628 | 82·1 | 1636 | 55·3 | 854 | 66·6 | 3330 | 77·0 | | 132 | | 80·0 | | 51 | | 83·6 | | 3180 | | 75·8 | | 78 | | 63·9 | | 68 | | 69·4 | |  |
| **Exposure to physical violence**  Yes | 3424 | 2·6 | 188 | 6·4 | 53 | 4·1 | 172 | 4·0 | | 13 | | 7·8 | | 5 | | 8·3 | | 179 | | 4·3 | | 4 | | 3·3 | | 6 | | 6·2 | | <0·001 |
| No | 139902 | 97·4 | 2769 | 93·6 | 1229 | 95·9 | 4153 | 96·0 | 154 | | 92·2 | | 55 | | 91·7 | | 4024 | | 95·7 | | 118 | | 96·7 | | 91 | | 93·8 | |  | |

^1^p values are for a test of means or equal proportions^. 2^Short version of the Warwick Edinburgh Mental Well-Being Scale (WEMWBS) ^3^Mental health based on the General Health Questionnaire (GHQ) used in 2018 and the Kessler-6 used in 2020. ^4^ Score of more than one on Problem Gambling Severity Index ^5^Any use of drugs during the last year.

**Supplementary Table 6. Associations between ethnic (including migrant and refugee) and gender identities and health and well-being in 164,118 individuals aged 16-84 years who answered the Swedish National Public Health Survey (2018-20). Estimates are from multiple logistic and linear regression models (models adjusted for sex at birth, age and educational level)**

| **Ethnic and gender identities** | **General health** | **Mental wellbeing (WEMWBS)^1^** | **Mental ill-health^2^** | **Suicidal ideation (lifetime)** | **Suicide attempts** | **Risk alcohol use** | **Risk gambling** | **Drug use** |
| --- | --- | --- | --- | --- | --- | --- | --- | --- |
|  | **OR (95% CI)** | **β (95% CI)** | **OR (95% CI)** | **OR (95% CI)** | **OR (95% CI)** | **OR (95% CI)** | **OR (95% CI)** | **OR (95% CI)** |
| **Number of yes** | 8352 | N/A | 16824 | 16286 | 4624 | 23856 | 4751 | 2930 |
| **White cisgender**  **(N=152827)** | Ref (1) | Ref (1) | Ref (1) | Ref (1) | Ref (1) | Ref (1) | Ref (1) | Ref (1) |
| **Number of yes** | 79 | N/A | 165 | 198 | 80 | 105 | 28 | 24 |
| **White transgender**  **(N=693)** | **3·13 (2·01–4·86)** | **-1·84 (-2·52–-1·16)** | **2·51 (1·82–**  **3·46)** | **4·34 (3·23–5·82)** | **3·37 (2·13–5·32)** | 0·94 (0·64–  1·37) | 1·48 (0·66–3·31) | 1·07 (0·54–  2·12) |
| **Number of yes** | 512 | N/A | 1038 | 596 | 257 | 240 | 394 | 87 |
| **Migrant cisgender**  **(N=5442)** | **1·48 (1·25–1·76)** | **-0·23 (-0·48–**  **-0·02)** | **1·54 (1·35–1·76)** | **0·74 (0·63–**  **0·87)** | 1·06 (0·83–1·35) | **0·19 (0·15–**  **0·24)** | **1·95 (1·58–2·40)** | **0·38 (0·26–**  **0·56)** |
| **Number of yes** | 11 | N/A | 22 | 15 | 16 | 6 | 19 | **1** |
| **Migrant transgender**  **(N=109)** | 1·18 (0·47–3·00) | 0·65 (-1·25–2·55) | 1·25 (0·53–2·94) | 1·17 (0·47–2·95) | **2·85 (1·05–7·74)** | 0·49 (0·15–  1·56) | 1·42 (0·72–2·82) | **0·07 (0·01–**  **0·56)** |
| **Number of yes** | 363 | N/A | 733 | 572 | 233 | 374 | 329 | 87 |
| **Refugee cisgender**  **(N=4991)** | **1·35 (1·12–1·63)** | -0·02 (-0·24–  0·20) | 1·11 (0·97–1·28) | 0·83 (0·71–  0·97) | 1·05 (0·82–1·34) | **0·42 (0·35–**  **0·50)** | **2·07 (1·69–2·54)** | **0·55 (0·38–**  **0·81)** |
| **Number of yes** | 5 | N/A | 8 | 14 | 12 | 8 | 6 | 3 |
| **Refugee transgender**  **(N=56)** | **4·24(1·01-17·87)** | 0·75 (-0·87-2·38) | 2·71 (0·89-8·23) | 1·90 (0·60-6·00) | 1·83 (0·69-4·86) | 1·17 (0·34-3·96) | **8·62 (1·94-38·40)** | 4·03 (0·92-17·73) |

^1^The Short Warwick-Edinburgh Mental Wellbeing Scale (WEMWBS) ^2^Kessler-6 and General Health Questionnaire (GHQ-5). Text in bold indicates 95% CI that do not include 1 (or zero)

**Supplementary table 7. Associations between ethnic (including migrant and refugee) and gender identities and experiences of violence in 164,118 individuals aged 16-84 years who answered the National Public Health Survey (2018-20). Estimates are from multiple logistic regression models (models adjusted for sex, age and educational level)**

| **Ethnic and gender identities** | **Physical violence** | **Threats** | **Discrimination** | **Any violence** |
| --- | --- | --- | --- | --- |
|  | **OR (95% CI)** | **OR (95% CI)** | **OR (95% CI)** | **OR (95% CI)** |
| **Number of yes** | 3753 | 5862 | 27923 | 31714 |
| **White cisgender**  **(N=152827)** | Ref (1) | Ref (1) | Ref (1) | Ref (1) |
| **Number of yes** | 57 | 72 | 215 | 266 |
| **White transgender**  **(N=693)** | **3·16 (1·86–5·36)** | **1·95 (1·26–3·02)** | **2·10 (1·54–2·86)** | **2·41 (1·79–3·25)** |
| **Number of yes** | 243 | 358 | 1158 | 1429 |
| **Migrant cisgender**  **(N=5442)** | **1·43 (1·14–1·81)** | 1·21 (0·99–1·49) | 0·99 (0·88–1·12) | 1·10 (0·98–1·23) |
| **Number of yes** | 19 | 14 | 21 | 39 |
| **Migrant transgender**  **(N=109)** | **6·31 (2·75–14·52)** | **2·97 (1·17–7·56)** | 0·95 (0·40–2·28) | **2·36 (1·20–4·63)** |
| **Number of yes** | 212 | 288 | 1188 | 1390 |
| **Refugee cisgender**  **(N=4991)** | **1·94 (1·54–2·46)** | **1·38 (1·11–1·70)** | **1·19 (1·06–1·34)** | **1·32 (1·18–1·48)** |
| **Number of yes** | 10 | 7 | 12 | 19 |
| **Refugee transgender**  **(N=56)** | **7·46 (2·97–18·70)** | 2·72 (0·91–8·07) | 1·44 (0·49–4·20) | 2·07 (0·81–5·32) |

Text in bold indicates 95% CI that do not include 1

**Supplementary Table 8. Associations between ethnic (including migrant and refugee) and sexual identities and health and well-being in 157,414 individuals aged 16-84 years who answered the Swedish National Public Health Survey (2018-20). Estimates are from multiple logistic and linear regression models (models adjusted for sex, age and education)**

| **Ethnic and sexual identities** | **General health** | **Mental wellbeing (WEMWBS)^1^** | **Mental ill-health^2^** | **Suicidal ideation (lifetime)** | **Suicide attempts** | **Risk alcohol use** | **Risk gambling** | **Drug use** |
| --- | --- | --- | --- | --- | --- | --- | --- | --- |
|  | **OR (95% CI)** | **β (95% CI)** | **OR (95% CI)** | **OR (95% CI)** | **OR (95% CI)** | **OR (95% CI)** | **OR (95% CI)** | **OR (95% CI)** |
| **Number of yes** | 7756 | N/A | 15175 | 14235 | 3819 | 22441 | 4274 | 2491 |
| **White heterosexual**  **(N=144127)** | Ref (1) | Ref (1) | Ref (1) | Ref (1) | Ref (1) | Ref (1) | Ref (1) | Ref (1) |
| **Number of yes** | 291 | N/A | 860 | 1276 | 514 | 685 | 97 | 303 |
| **White bisexual**  **(N=2967)** | **2·49 (1·96–3·16)** | **-1·88 (-2·24–**  **-1·52)** | **2·32 (1·99–2·71)** | **3·92 (3·41–4·52)** | **4·02 (3·30–4·90)** | 1·17 (0·99–1·38) | 0·94 (0·61–1·43) | **2·54 (2·0–3·22)** |
| **Number of yes** | 90 | N/A | 245 | 340 | 124 | 288 | 52 | 66 |
| **White homosexual**  **(N=1288)** | **1·89 (1·25–2·84)** | 1·15 (0·70–1·61) | **1·86 (1·44–2·39)** | **2·62 (2·07–3·31)** | **4·02 (2·86–5·64)** | 1·21 (0·95–1·56) | **1·76 (1·06–2·90)** | 0·90 (0·57–  1·43) |
| **Number of yes** | 373 | N/A | 802 | 465 | 193 | 185 | 285 | 72 |
| **Migrant heterosexual**  **(N=4356)** | **1·55 (1·28–1·89)** | **-0·40 (-0·89–**  **-0·12)** | **1·56 (1·34–1·81)** | **0·79 (0·66–0·95**) | 1·13 (0·86–1·48) | **0·19 (0·15–**  **0·24)** | **1·81 (1·42–2·29)** | **0·43 (0·29–**  **0·65)** |
| **Number of yes** | 11 | N/A | 38 | 29 | 13 | 12 | 17 | 5 |
| **Migrant bisexual**  **(N=168)** | 0·58 (0·27–1·28) | 0·20 (-0·97–  1·37) | 1·81 (0·93–3·50) | 1·32 (0·62–2·82) | 2·11 (0·65–6·87) | **0·30 (0·10–**  **0·88)** | **2·77 (1·17–6·57)** | 0·36 (0·11–  1·22) |
| **Number of yes** | 6 | N/A | 15 | 14 | 7 | 4 | 8 | 9 |
| **Migrant homosexual**  **(N=61)** | 2·95 (0·68–12·82) | 0·12 (-2·54–2·77) | 2·81 (0·99-7·92**)** | 1·48 (0·52–4·23) | 3·31 (0·66–16·45) | **0·10 (0·03–0·29)** | 1·05 (0·36–3·09) | 3·25 (0·89–11·83) |
| **Number of yes** | 279 | N/A | 574 | 479 | 182 | 319 | 248 | 72 |
| **Refugee heterosexual**  **(N=4226)** | **1·36 (1·10–1·69)** | -0·07 (-0·30–  0·16) | 1·10 (0·94–1·28) | 0·87 (0·73–1·03) | 1·18 (0·90-1·54) | **0·41 (0·33–0·49)** | **1·89 (1·50–2·37)** | **0·51 (0·34-0·76)** |
| **Number of yes** | 9 | N/A | 30 | 39 | 16 | 11 | 4 | 8 |
| **Refugee bisexual**  **(N=123)** | 2·62 (0·89–7·74) | -0·60 (-1·67–0·46) | 1·47 (0·70–3·09) | **3·82 (1·9–7·68)** | 2·65 (0·93–7·58) | 0·69 (0·25–1·90) | 1·44 (0·27–7·73) | 1·83 (0·48–6·96) |
| **Number of yes** | 6 | N/A | 19 | 19 | 10 | 16 | 7 | 3 |
| **Refugee homosexual**  **(N=98)** | 1·01 (0·38–2·72) | 0·01 (-1·64–1·66) | 1·37 (0·63–2·98) | 1·39 (0·59–3·23) | 1·40 (0·60–3·23) | 0·83 (0·34–2·04) | 3·08 (0·93–10·18) | 1·30 (0·25–6·68) |

^1^ The Short Warwick-Edinburgh Mental Wellbeing Scale (WEMWBS) ^2^ Kessler-6 and General Health Questionnaire (GHQ-5) Text in bold indicates 95% CI that do not include 1

**Supplementary Table 9. Associations between ethnic (including migrant and refugee) and sexual identities and experiences of violence in 157,414 individuals aged 16-84 years who answered the National Public Health Survey (2018-20) Estimates are from multiple logistic regression models (models adjusted for sex, age, and educational level)**

| **Ethnic and sexual identities** | **Physical violence** | **Threats** | **Discrimination** | **Any violence** |
| --- | --- | --- | --- | --- |
|  | **OR (95% CI)** | **OR (95% CI)** | **OR (95% CI)** | **OR (95% CI)** |
| **Number of yes** | 3424 | 5346 | 25589 | 29190 |
| **White heterosexual**  **(N=144127)** | Ref (1) | Ref (1) | Ref (1) | Ref (1) |
| **Number of yes** | 188 | 304 | 1321 | 1415 |
| **White bisexual**  **(N=2967)** | **1·88 (1·41–2·52)** | **1·88 (1·50–2·36)** | **2·25 (1·95–2·60)** | **2·18 (1·90–2·51)** |
| **Number of yes** | 53 | 103 | 428 | 469 |
| **White homosexuals**  **(N=1288)** | 1·26 (0·72–2·18) | **1·55 (1·05–2·29)** | **2·15 (1·72–2·69)** | **1·97 (1·58–2·44)** |
| **Number of yes** | 172 | 280 | 992 | 1190 |
| **Migrant heterosexual**  **(N=4356)** | 1·29 (0·99–1·69) | **1·26 (1·01–1·58)** | 1·12 (0·98–1·29) | **1·22 (1·08–1·39)** |
| **Number of yes** | 13 | 15 | 33 | 50 |
| **Migrant bisexuals**  **(N=168)** | **2·71 (1·08–6·81)** | 1·82 (0·70–4·78) | 1·06 (0·53–2·10) | 1·35 (0·76–2·37) |
| **Number of yes** | 5 | 7 | 10 | 16 |
| **Migrant homosexuals**  **(N=61)** | 3·41 (0·68–17·10) | 2·49 (0·61–10·16) | **0·39 (0·16–0·98)** | 0·79 (0·27–2·30) |
| **Number of yes** | 179 | 222 | 1014 | 1187 |
| **Refugee heterosexual**  **(N=4226)** | **2·08 (1·62–2·67)** | **1·26 (1·00–1·60)** | **1·24 (1·09–1·41)** | **1·40 (1·24–1·59)** |
| **Number of yes** | 4 | 9 | 44 | 46 |
| **Refugee bisexuals**  **(N=123)** | 1·72 (0·34–8·64) | 1·98 (0·72–5·48) | **2·28 (1·17–4·47)** | **2·37 (1·22–4·59)** |
| **Number of yes** | 6 | 8 | 30 | 34 |
| **Refugee homosexuals**  **(N=98)** | 3·11 (0·86–11·17) | 2·50 (0·80–7·84) | 1·52 (0·70–3·31) | 1·83 (0·89–3·75) |

Text in bold indicates 95% CI that do not include 1
